# Perceived stress and life satisfaction among elderly migrants in China: A moderated mediation model

**DOI:** 10.3389/fpsyg.2022.978499

**Published:** 2022-08-15

**Authors:** Yanjie Hou, Shiyuan Yan, Lin Zhang, Hao Wang, Ruyue Deng, Wenjing Zhang, Jun Yao

**Affiliations:** ^1^School of Health Policy and Management, Nanjing Medical University, Nanjing, China; ^2^School of Nursing, Nanjing Medical University, Nanjing, China; ^3^Institute of Healthy Jiangsu Development, Nanjing Medical University, Nanjing, China

**Keywords:** perceived stress, life satisfaction, anxiety, resilience, elderly migrants

## Abstract

**Objective:**

Our study aims to test whether anxiety mediated the association between perceived stress and life satisfaction and whether the mediating effect was moderated by resilience among elderly migrants in China.

**Methods:**

We used self-reported data collected from 654 elderly migrants in Nanjing. Regression analyses using bootstrapping methods were conducted to explore the mediating and moderating effects.

**Results:**

The results showed that anxiety mediated the relationship between perceived stress and life satisfaction (indirect effect = –0.040, CI [–0.066, –0.017]). Moreover, moderated mediated analysis indicated that resilience moderated the path between anxiety and life satisfaction (moderating effect = 0.034, 95% CI [0.021, 0.048]). In particular, anxiety had a negative impact on life satisfaction only for Chinese elderly migrants with lower resilience.

**Conclusion:**

Our study suggests that perceived stress could reduce life satisfaction among elderly migrants as their anxiety levels increase. Fortunately, elderly migrants’ resilience could undermine this negative effect.

## Introduction

The 7th National Census Bulletin issued by the National Bureau of Statistics shows that China’s floating population has reached 376 million in 2021, and predicts that the number of elderly migrants will continue to grow (Li and Dou, 2022). Facing the dual dilemma of aging and mobility, elderly migrants may have poor adaptability (Alizadeh-Khoei et al., 2011). Some studies indicated that immigrants had higher distress than the native population at the initial stage of their immigration process (Chen, 2011). After moving to new living environments, elderly migrants face a variety of problems due to the change in social networks (Dietzel-Papakyriakou and Olbermann, 1996), longstanding household registration (“hukou”) (Hou et al., 2021)policy restrictions, cultural differences, living styles, and language barriers. Due to these objective socio-economic disadvantages, migrant older adults are easy to feel a sense of relative deprivation (Xia and Ma, 2020). As a result, a series of mental health problems have emerged, such as increased perceived stress, and increased anxiety, ultimately leading to a decline in life satisfaction.

Life satisfaction is a component of subjective wellbeing (Diener, 1996) and is a cognitive judgment of an individual’s satisfaction with his or her entire life. It is one of the most important factors affecting the mental health of an individual and determining his/her adaptation to old age (Tambag, 2013). Improving the life satisfaction of migrant older adults contributes to their successful aging and improving quality of life. Previous studies have revealed that personal variables, such as perceived stress (Chen et al., 2017), anxiety (Brailovskaia et al., 2018), and resilience (Azpiazu Izaguirre et al., 2021), could strongly predict individuals’ life satisfaction, but there is no research to confirm their combined impact on life satisfaction. How to make the huge number of elderly migrants have a happy life in their old age is a major matter with long-term and far-reaching significance. Therefore, it is necessary to explore the influence of the psychological factors that regulate or intervene in the life satisfaction of migrant older adults. The purpose of this study is to investigate the relationship between perceived stress, anxiety, and life satisfaction of elderly migrants, and to investigate the mediating role of anxiety in the relationship between perceived stress and life satisfaction of elderly migrants, as well as the moderating role of resilience.

### Perceived stress and life satisfaction

Perceived stress is a psychological reaction generated after an individual experiences stimuli in the environment and evaluates them cognitively (Cohen et al., 1983). When an individual encounters frustration and pressure, it does not necessarily directly affect the individual itself. Only the perceived press can produce a series of emotional and behavioral reactions. With irreversible changes in physiological functions, the elderly become more sensitive to their surroundings and have a stronger awareness of external pressure events. Even trivial things in life are prone to strong emotional and emotional responses. Chronic diseases, bereavement, and sleep disorders are common sources of stress. Compared with the general elderly, the elderly migrants are more likely to suffer from intergenerational conflict, economic source instability, loss of social circle, and other aspects of pressure. They often have difficulty responding effectively to negative events and are prone to perceive higher levels of external stress. The concept of perceived stress has been extensively researched since its inception (McHugh et al., 2020; Moseley et al., 2021). Nonetheless, there has been little focus on elderly migrants’ perceived stress.

Life satisfaction is a cognitive process in which individuals evaluate their state of pleasure in life by comparing their current circumstances to a range of personal ideal criteria (Lee et al., 2016). Life satisfaction is an essential metric for assessing the physical and mental health, as well as the wellbeing, of older adults (Van Damme-Ostapowicz et al., 2021), which is of great significance for promoting active aging (Gana et al., 2013). According to Selye’s stress theory, a high or prolonged stress level will consume an individual’s resources and psychological energy to cope with stress, and reduce life satisfaction (Randall and Bodenmann, 2009). Existing research revealed that perceived stress was a well-known cause of numerous emotional issues, including depression (Das et al., 2020), loneliness (Peavy et al., 2022), and anxiety (Yu and Liu, 2018). And perceived stress is a significant forecaster of low life satisfaction (Kim, 2019). Existing researches indicate that perceived stress has a significantly predictive impact on life satisfaction (Hamarat et al., 2001; Hwang and Hyeseong, 2020). The greater the perceived stress, the lower life satisfaction. Therefore, perceived stress may be one of the factors affecting life satisfaction among seniors. Thus, in this study, we focused on Chinese elderly migrants’ perceived stress and hypothesized that their perceived stress would be negatively correlated to their life satisfaction.

### Anxiety, perceived stress, and life satisfaction

Anxiety in later life is a very common mental illness (Baxter et al., 2013; El-Gabalawy et al., 2013). As the number of seniors increases globally, anxiety will become a prevalent problem in later life (Balsamo et al., 2018). Some studies indicated anxiety disorders, depression, and stress prevail among the elderly (Babazadeh et al., 2016). Previous literature has shown a significant positive correlation between perceived stress and anxiety (Tan et al., 2021). Older adults with higher perceived stress have higher levels of anxiety (Shi et al., 2020). People are prone to anxiety symptoms such as agitation after experiencing a period of stressful states and without a reasonable release of stress. Moreover, anxiety is negatively correlated to life satisfaction (Yu et al., 2020; Mei et al., 2021; Hoseini-Esfidarjani et al., 2022). Anxiety, according to a previous study, is a possible cause that reduces the wellbeing in seniors (Brown Wilson et al., 2019). Long-term anxiety has a significant impact on the health of the seniors, causing not only a series of symptoms such as depression (Kandola and Stubbs, 2020), but also a series of physical conditions, such as decreased sleep function, hypertension, and cognitive decline (Andreescu and Lee, 2020), which can jeopardize physical and psychological health and reduce life satisfaction (Abreu et al., 2018; Weger and Sandi, 2018). Therefore, anxiety may operate as a mediator in the relationship between perceived stress and life satisfaction.

Migration is an extremely stressful phenomenon that produces significant levels of anxiety and depressive symptoms (Bhugra, 2004; Levecque et al., 2007; Familiar et al., 2011). Previous studies in China have identified a significant increase in the prevalence of anxiety and depression among elderly migrants, which has become an important issue affecting their quality of life (Yu and Liu, 2018; Li and Kong, 2022). Compared with the local elderly, elderly migrants face low levels of social interaction (Lin et al., 2020), and lack of contact with friends. They are separated from society (Kim et al., 2012), then lose their original social capital and face communication barriers (Liu et al., 2013). They lack channels for dealing with perceived stress, leading them to easily dwell on pessimistic ruminations and eventually develop mental disorders, such as depression (Kim et al., 2012) and loneliness (Tang et al., 2020). In addition, the elderly with rural household registration, chronic disease, no pension insurance or pension, and no company with spouse may experience stronger culture shock, poorer physical health, poorer economic status, lack of emotional support from spouse, and are more likely to fall into anxiety. When migrant older adults are in a state of chronic anxiety, they tend to be less interested in life, which ultimately leads to a decrease in life satisfaction. As a result, we further predicted anxiety as a mediator in the relationship between perceived stress and life satisfaction among Chinese elderly migrants.

### Resilience, anxiety, and life satisfaction

Not all anxious individuals report decreased life satisfaction. Some beneficial factors may have contributed to the prevention of anxiety turning into depressive symptoms. The American Psychological Association considers resilience as a person’s capacity to recover from stressful conditions and as a good adaptation to traumatic events (Montazeri, 2008). In accord with the resilience framework, resilience can act as a moderating buffer against the harmful effects of adversity as a dynamic process (Kumpfer, 2002). Moreover, resilience is being studied as a source of support for life satisfaction (Plexico et al., 2019). Many researches have found a link between resilience and life satisfaction (Azpiazu Izaguirre et al., 2021; Sørensen et al., 2021). Furthermore, a study discovered that nurses with high resilience were prone to experience decreased anxiety (Labrague and De Los Santos, 2020). However, insufficient resilience may contribute to negative mental health indicators (e.g., anxiety) (Hu et al., 2015; Wollny and Jacobs, 2021). It is difficult for elderly migrants who lack resilience to get back fast from stress, adjust successfully, keep excellent mental health, or diminish adversity and tackle problems, thus falling into the negative emotion of anxiety. Based on the above theoretical framework and findings, we hypothesized that resilience would moderate the association between anxiety and life satisfaction. Moreover, anxiety had a negative impact on life satisfaction only for Chinese elderly migrants with lower resilience.

### Hypothetical research model

**FIGURE 1:**
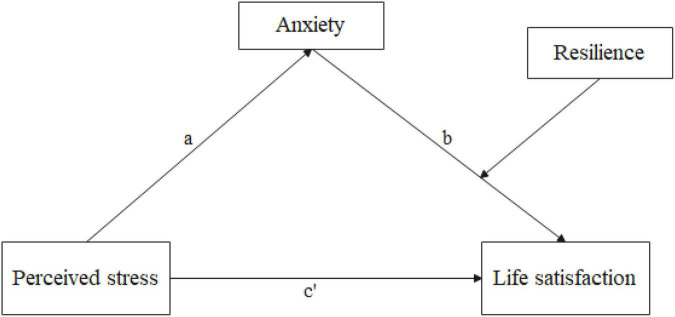
The hypothesized moderated mediation model.

We concentrated on the impact of perceived stress on life satisfaction among Chinese elderly migrants in this study. Furthermore, we investigated the mechanism of this association using anxiety as a mediator and resilience as a moderator. We proposed the following hypotheses (Figure 1):

1. Chinese elderly migrants’ perceived stress would be negatively correlated to their life satisfaction.

2. Chinese elderly migrants’ anxiety would mediate the relationship between perceived stress and their life satisfaction.

3. The indirect impact of perceived stress on life satisfaction through anxiety would be dependent on resilience. In particular, anxiety had a negative impact on life satisfaction only for Chinese elderly migrants with lower resilience.

## Measures

### Participants and procedures

The data in this study came from the Social Science Foundation Project of People’s Republic of China “A follow-up study on the mechanism of intergenerational relationship on the mental health of elderly migrants.” This project was performed from September 2019 to September 2020 in Nanjing, Jiangsu Province, China. The project firstly randomly selected 7 districts in Nanjing (Qinhuai, Qixia, Gulou, Xuanwu, Jianye, Yuhuatai, and Jiangning District), then randomly selected 3 communities in each district, and finally recruited elderly migrants who met the inclusion criteria in these 21 communities. All participants were told about the study’s purpose and volunteered to take part. A standardized questionnaire was used to conduct face-to-face interviews with all participants. All interviewers had medical research backgrounds and received a uniform and standardized training before the project. This study used the first phase survey data of the project. Inclusion criteria were: (1) age ≥ 60 years old; (2) household registration not moved to Nanjing; (3) moved to Nanjing ≤ 10 years. Power analysis for a multiple regression was conducted in G*power to determine a sufficient sample size using an alpha of 0.05, a power of 0.95, and a medium effect size (*f*^2^ = 0.15) (Faul et al., 2009). Based on the aforementioned assumptions, the desired sample size was 229–250 (with the consideration for 10–20% of uncompleted surveys). Following screening, 654 people were chosen for this research finally. The sample size of this study is adequate.

### Instruments

#### Life satisfaction

The elderly migrants’ life satisfaction was measured using the Satisfaction with Life Scale (SWLS) (Diener et al., 1985). SWLS contains five items. Each item is rated on a scale of 1 (strongly disagree) to 7 (strongly agree), with a total score ranging from 5 to 35. Higher scores reflect higher life satisfaction. The Chinese version has been widely used to measure the life satisfaction of the elderly, with satisfactory reliability and validity (Tian and Wang, 2021). Cronbach’s alpha for the present sample was 0.887. In the CFA, GFI = 0.946. This questionnaire is reliable and valid for the measurement of life satisfaction among the elderly migrants.

#### Perceived stress

The elderly migrants’ perceived stress over the past month was assessed using the Perceived Stress Scale (PSS) (Cohen et al., 1983). PSS is made up of 14 items and two sub-dimensions: the feeling of being out of control and nervousness. Items 4, 5, 6, 7, 9, 10, and 13 belong to the uncontrollable dimension and are scored in reverse. Items 1, 2, 3, 8, 11, 12, and 14 belong to the nervous dimension. Each item is rated on a scale of 1 (never) to 4 (always), with a total score ranging from 0 to 56. The Chinese version is translated by Yang and has high reliability and validity (Yang and Huang, 2003). Higher scores imply a greater level of perceived stress. Cronbach’s alpha for the present sample was 0.809. In the CFA, GFI = 0.717. This questionnaire is reliable and valid for the measurement of perceived stress among the elderly migrants.

#### Anxiety

The elderly migrants’ anxiety was assessed using the Hospital Anxiety and Depression Scale-Anxiety (HADS-A) (Zigmond and Snaith, 1983). The 7-item scale measured if participants currently experienced the following symptoms. It includes questions like “I feel nervous or excited” and “The concern thoughts always hovering in my mind” on a 4-point scale (1 = not at all, 4 = most of the time). The total score results in a score between 7 and 28. The higher the score, the higher the sense of anxiety. Cronbach’s alpha for the present sample was 0.787. In the CFA, GFI = 0.890. This questionnaire is reliable and valid for the measurement of anxiety among the elderly migrants.

#### Resilience

The elderly migrants’ resilience was measured using the 10-item Connor–Davidson Resilience Scale (CD-RISC-10) (Campbell-Sills and Stein, 2007) the scale contains 10 items. Each item is rated on a scale of 1 (never) to 5 (always), with a total score ranging from 0 to 50. Several studies have shown that CD-RISC-10 has high reliability and validity in the Chinese population (Zhang et al., 2021; Hu et al., 2022). Higher scores imply a greater level of resilience. Cronbach’s alpha for the present sample was 0.924. In the CFA, GFI = 0.935. This questionnaire is reliable and valid for the measurement of resilience among the elderly migrants.

#### Sociodemographic characteristics

Age (in years), gender (0 = female, 1 = male), marital status (0 = single/divorced/widowed, 1 = married), education background (1 = primary school or lower, 2 = middle or high school, 3 = college or above), yearly income (< 5,000 RMB, 5,000 RMB–10,000 RMB, 10,000 RMB–40,000 RMB, > 40,000 RMB), self-reported physical health (from 1 = very unhealthy to 5 = very healthy), duration of migration (in years), social support, and social participation were all control variables.

### Data analysis

In this study, we use SPSS25.0 to measure the sample characteristic, the descriptive statistics, and the Pearson correlation analysis. To assess the importance of the mediated models, we used the bootstrapping procedure in Hayes’ PROCESS macro program. According to Mackinnon et al. (2004), we utilized Model 4 to test the indirect influence of perceived stress on life satisfaction through anxiety. Moreover, we tested the moderated mediation in this study by Hayes’s PROCESS macro Model 14. We predicted resilience to moderate the relationship between anxiety and life satisfaction, with the indirect effect depending on resilience level (Hypothesis 3). The bootstrap confidence intervals (CIs) determine whether the effects in Model 4 and Model 14 are significantly based on 5,000 random samples (Hayes and Scharkow, 2013). An effect is regarded as significant if the CIs do not include zero.

## Results

### Common method biases test

Using Harman’s one-way test for all questions on the four scales, it was found that the first common factor analyzed explained only 32.38% (< 40%) of the variance, indicating that there was no serious common method bias in this study despite the use of the questionnaire.

### Descriptive data and Pearson correlations

The sociodemographic characteristics were shown in Table 1. 654 elderly migrants had an average age of 66.08 ± 4.67 (range: 60–86) years, with an average of 3.96 ± 1.46 (range: 0.5–5.5) years of migration. The average level of social support is 22.30 ± 5.02 (range: 9–40) and the average level of social participation is 39.08 ± 5.56 (range: 22–48). Most elderly migrants were females (67.0%), married (84.6%), reported primary school or lower (54.1%) and had a fair physical health status (65.4%). The yearly income of most elderly migrants was less than 5,000 RMB (38.1%).

**TABLE 1:** Sociodemographic information of the participants (*n* = 654).

Variable	Mean ± SD (range) *N* (%)
Age	66.05 ± 4.67 (60–86)
Duration of migration	3.96 ± 1.46 (0.5–5.5)
Social support	22.30 ± 5.02 (9–40)
Social participation	39.08 ± 5.56 (22–48)
**Gender**	
Male	216 (33.0%)
Female	438 (67.0%)
**Marital status**	
Single/divorced/widowed	101 (15.4%)
Married	553 (84.6%)
**Education background**	
Primary school or below	354 (54.1%)
Middle or high school	262 (40.1%)
college or above	38 (5.8%)
**Yearly income**	
<5,000 RMB	249 (38.1%)
5,000 RMB–10,000 RMB	126 (19.3%)
10,000 RMB–40,000 RMB	199 (30.4%)
>40,000 RMB	80 (12.2%)
**Self-reported physical health**	
Poor	183 (28.0%)
Fair	428 (65.4%)
Good	43 (6.6%)

Table 2 presented the Pearson correlations of the study variables. The results indicated that perceived stress (*r* = –0.516, *p* < 0.01), anxiety (*r* = –0.378, *p* < 0.01), resilience (*r* = 0.514, *p* < 0.01) were related to life satisfaction. Perceived stress was positively correlated with anxiety (*r* = 0.499, *p* < 0.01) and negatively correlated with resilience (*r* = –0.607, *p* < 0.01). Anxiety was negative correlated to resilience (*r* = –0.378, *p* < 0.01). Thus, Hypothesis 1 was verified.

**TABLE 2:** Bivariate correlation among perceived stress, anxiety, resilience, and life satisfaction (*n* = 654).

	M ± SD	1	2	3	4
1. Perceived stress	20.87 ± 7.66	1			
2. Anxiety	11.20 ± 3.36	0.499**	1		
3. Resilience	33.06 ± 7.53	–0.607**	–0.378**	1	
4. Life satisfaction	26.74 ± 5.37	–0.516**	–0.378**	0.514**	1

**p < 0.01.

### Mediation analyses

Table 3 showed the indirect impact of perceived stress on life satisfaction *via* the anxiety. After we controlled for the effects of age, education, etc. the indirect mediated effect of perceived stress on life satisfaction was significant (β = –0.040, CI [–0.066, –0.017]). Perceived stress was a significantly negative predictor on life satisfaction (β = –0.284, CI [–0.337, –0.231]). Perceived stress was a significantly positive predictor of anxiety (β = 0.184, CI [–0.150, –0.218]), while anxiety had a significantly negatively predicted effect on life satisfaction (β = –0.219, CI [–0.338, –0.100]). Moreover, the direct effect of perceived stress on life satisfaction was still significant (β = –0.244, CI [–0.301, –0.187]) when mediating variables were added. Additionally, the upper and lower bounds of the bootstrap 95% CI for the direct effect of perceived stress on life satisfaction and the mediating effect of anxiety did not include 0, indicating that the mediating effect was significant. The mediation effect accounted for 14.08% of the total effect, which confirmed that anxiety played a partial mediating role in the relationship between perceived stress and life satisfaction. Thus, Hypothesis 2 was verified.

**TABLE 3:** Mediation effect of anxiety on the relationship between perceived stress and life satisfaction (*n* = 654).

Outcome	Mediation analysis paths	Estimated	95% bias-corrected CI		Proportion
			LLCI	ULCI	
SWLS	Total effect	–0.284***	–0.337	–0.231	
	Direct effect	–0.244***	–0.301	–0.187	85.92%
	Indirect effect	–0.040***	–0.066	–0.017	14.08%
	PSS→Anxiety	0.184***	0.150	0.218	
	Anxiety→SWLS	–0.219***	–0.338	–0.100	

Controlling for age, duration of migration, gender, marital status, education background, yearly income, self-reported physical health, and social support, social participation. SWLS, Life Satisfaction, PSS, Perceived Stress. ***p < 0.001.

### Testing for the moderated mediation

**FIGURE 2:**
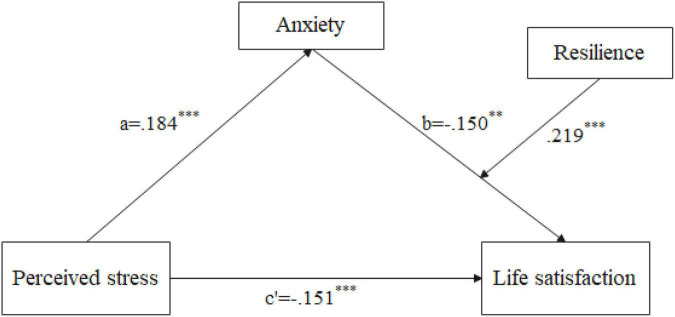
The final moderated mediation model (^∗∗^*p* < 0.01; ^∗∗∗^*p* < 0.001).

**FIGURE 3:**
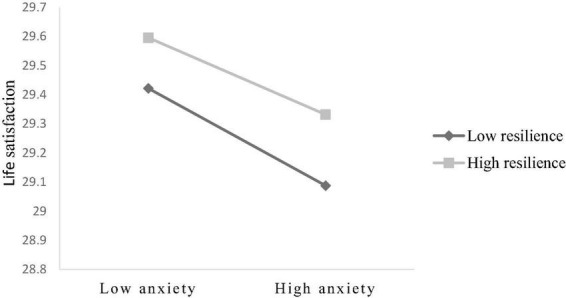
The moderating effect of resilience on the relation between anxiety and life satisfaction.

Hypothesis 3 predicted that resilience would moderate the indirect effect in Hypothesis 2. The final moderated mediation model is shown in Figure 2. The indexes of moderated mediation were significant (see Table 4; β = 0.006, 95% CI [0.004, 0.009]), which showed that the indirect effect of perceived stress on life satisfaction *via* the anxiety varied with the level of resilience. The interaction term anxiety × resilience was significant in the moderated mediation model (see Table 4; β = 0.034, 95% CI [0.021, 0.048]). We graphed the interaction effects in Figure 3 to help explain the interaction outcome. When compared to elderly migrants with high resilience, anxiety had a greater impact on life satisfaction among elderly migrants with poor resilience. Moreover, we showed the indirect impact of the moderator at different values (1 SD below the mean, the mean, and 1 SD above the mean). The indirect effects were significant for elderly migrants with low and average levels of resilience (see Table 5; effect_lowlevel_ = –0.409, *t* = –5.549, and *p* < 0.001; effect_averagelevel_ = –0.150, *t* = –2.599, and *p* < 0.01), whereas for elderly migrants with higher resilience, the indirect effect was not significant (see Table 5; effect_highlevel_ = 0.109, *t* = 1.324, and *p* > 0.1). Thus, Hypothesis 3 was verified.

**TABLE 4:** The moderated mediation model with anxiety as a mediator and resilience as a moderator (*n* = 654).

Variable	Life satisfaction
Index of moderated mediation[95% CI]	0.006 [0.004, 0.009]
**Direct effects**	
Perceived stress	–0.151 [–0.211, –0.090]
Anxiety	–0.150 [–0.263, –0.037]
Resilience	0.219 [0.162, 0.276]
Anxiety × Resilience	0.034 [0.021, 0.048]
*R* ^2^	0.270
F	23.825***

Controlling for age, duration of migration, gender, marital status, education background, yearly income, self-reported physical health, and social support, social participation.

***p < 0.001.

**TABLE 5:** Conditional effects of anxiety on life satisfaction at values of resilience.

	*Effect*	*t*	LLCI	ULCI
Low resilience	–0.409	–5.549***	–0.554	–0.264
Average resilience	–0.150	–2.599**	–0.263	–0.037
High resilience	0.109	1.324	–0.053	0.271

Controlling for age, duration of migration, gender, marital status, education background, yearly income, self-reported physical health, and social support, social participation.

**p < 0.01, ***p < 0.001.

## Discussion

The study’s findings revealed that Chinese elderly migrants’ perceived stress was negative relation to their life satisfaction (Hypothesis 1), and anxiety mediated this connection (Hypothesis 2). Furthermore, anxiety only mediated the relationship between perceived stress and life satisfaction among Chinese elderly migrants with lower resilience (Hypothesis 3).

### Perceived stress and life satisfaction

First, perceived stress was shown to have a substantial influence on the life satisfaction of elderly migrants in China, according to the results of this research, which is consistent with the previous studies showing high perceived stress could lead to poor life satisfaction (Kim, 2019; Xu et al., 2021; Ye et al., 2021; Young and Ah, 2021). In general, elderly migrants in China are more likely to experience stressful events such as concerns about their children’s situation, financial entanglements among family members, and worries about their poor health when they leave their familiar hometowns for reasons such as employment, caring for children or grandchildren, and retirement (Tang et al., 2020). The change of living environment may expose elderly migrants to environmental discomfort, cultural barriers, monotonous spiritual life, low community participation, low identification with urban communities, and the limited access to health service utilization (Song et al., 2017), further increasing perceived stress and causing the accumulation of negative emotions. The pressures of caring for offspring, household chores, and language barriers reduce the leisure time, recreational time, and social participation of elderly migrants. Many elderly migrants are silent and depressed due to the lack of friends and social interaction and even suffer from negative mental outcomes such as loneliness (Olofsson et al., 2021), ultimately causing a decline in life satisfaction.

### The mediating role of anxiety

As hypothesized, this study indicated that anxiety mediated the relationship between perceived stress and life satisfaction, perhaps revealing the underlying mechanism concerning how perceived stress influences life satisfaction indirectly. On the one hand, the primary purpose of mobility for most Chinese migrant older adults is to care for their grandchildren. The caregiver role is burdensome, and perceived stress occurs with increased workload and psychological demands. Caregivers may struggle to balance their caregiving duties with other aspects of their lives but remain silent about the perceived stress, aggravating their mental health and leading to anxiety symptoms (Shi et al., 2020). On the other hand, as they age, elderly migrants experience a further decline in cognitive abilities (Deary et al., 2009) and daily activities, have varying degrees of deteriorating health, and begin to require the care of children, which leads to increased perceived burdensomeness (Jahn et al., 2011). In addition, elderly migrants’ financial resources in their later life are mainly supported by their children (Wu et al., 2005), which to some extent brings a psychological burden to migrant older parents (Shenk, 2001)and endangers sentiments of dependency and lack of autonomy (Silverstein et al., 1996). These dilemmas have led to significantly higher levels of perceived stress among elderly migrants. They lack friends and social interactions (Lin et al., 2020), so that they are unable to dissipate perceived stress and develop psychological problems such as anxiety. Life satisfaction reflects a person’s spiritual and material life level, and anxiety is an important factor affecting the spiritual life level. If a migrant older adult has a high sense of anxiety, his evaluation of his spiritual and material living standards will be relatively low, and his life satisfaction will be further reduced. According to the analysis results, we propose that decreasing elderly migrants’ anxiety was a valid method to improve their life satisfaction. Previous research referred that migrants with poor subjective health status were possible to experience anxiety (Harjana et al., 2021) and reported that physical activity was a favorable element against anxiety among older adults (de Oliveira et al., 2019). And abundant social activities and emotional support from neighbors and friends can help cope with the monotony of daily life and relieve anxiety. Thus, elderly migrants should strengthen physical exercise, improve health self-efficacy, increase communication with family and friends to reduce anxiety levels and ultimately improve their life satisfaction.

### The moderating role of resilience

The present study also examined whether resilience moderated the association between anxiety and life satisfaction among elderly migrants in China. Resilience was discovered to be a facilitator of life satisfaction among elderly migrants, which supports the previous findings (Foster et al., 2019). Especially, this study discovered that resilience moderated the connection between anxiety and life satisfaction of elderly migrants. For elderly migrants with a lower level of resilience, anxiety might have a major influence on their life satisfaction, whereas, for those with a higher resilience level, it has no significant impact. Resilience has an important role in promoting the mental development of individuals, through the buffering effect, challenging effect and compensating effect, reducing anxiety symptoms, enhancing the strength of individuals when experiencing difficulties and adverse events, stimulating the potential of individuals (Li and Sun, 2014). Elderly migrants with more resilience have an optimistic view of life, are more psychologically healthy (Schoenmakers et al., 2017), maintain moderate communication and exchange with family members, and receive timely emotional support, respect, and help (Cao and Liang, 2020). The feeling of resilience might reduce older adults’ likelihood of indulging in anxiety. Therefore, this would make anxiety’s effect on life satisfaction non-significant. However, elderly migrants who lack resilience have insufficient psychological and social resources available, lack problem-solving ability and confidence. As a result, when they indulged in anxiety, they lacked the psychological resources to help them recover quickly and cope successfully, ultimately leading to a decrease in life satisfaction. It can be concluded that anxiety is a risk factor for reducing life satisfaction of the elderly migrants with lower resilience when compared to those with greater resilience. In future research and practical work on the life satisfaction of elderly migrants, resilience construction needs to be strengthened. It is necessary to further strengthen family and social support, increase protective resources against stress, and improve the adaptability and sense of belonging of elderly migrants. It is suggested that communities should actively carry out cultural activities, emphasize and pay attention to the cultural aging of the elderly (Du et al., 2018), and help to make more friends, which is conducive to timely emotional support for the elderly migrants, reduce anxiety, enhance the sense of belonging, and achieve higher life satisfaction through positive social relationships.

## Limitations and future studies

Some limitations should be improved in a future study. First of all, multiple assessment methods need to be considered to lower the subjective impact of self-report measures. Secondly, a cross-sectional survey was used in our paper, and the results could not determine causal inferences. In the future, additional research about the associations between perceived stress and life satisfaction could be undertaken, such as longitudinal studies. Furthermore, other variables could be dug out to explain the underlying psychological mechanisms between perceived stress and life satisfaction, such as loneliness, depression, social participation, self-efficacy, and so on. In addition, because this study used a questionnaire, older adults who were able to communicate smoothly with the interviewer and had no mobility impairment were enlisted to take part in this study. Future research should consider using other approaches to survey elderly migrants with cognitive impairments and limited mobility in daily activities in order to generalize the results to a larger population of elderly migrants.

## Implications

In summary, this was one of a few studies testing the mediating and moderating effects of anxiety and resilience on the relationship between perceived stress and life satisfaction among elderly migrants in China. Our findings not only suggested that elderly migrants’ life satisfaction could be affected by perceived stress through anxiety. This study also found that resilience could moderate the path way of anxiety to life satisfaction thus alleviating the indirect impact of perceived stress on life satisfaction, and anxiety had a negative impact on life satisfaction only for Chinese elderly migrants with lower resilience.

## Conclusion

Given these conclusions, we make the following recommendations. First, helping elderly migrants relieve perceived stress and anxiety is needed. On the one hand, when elderly migrants are caught in high perceived stress and anxiety, family and friends should give them enough emotional support (Liu et al., 2021). Teach them to release stress appropriately and listen carefully to their needs. On the other hand, more incentives or promotions should be arranged for elderly migrants to participate in these local social activities, such as community sports and physical exercise (Gao et al., 2014). As prior study (Tang et al., 2020) discovered that local friends accumulated social capital for migrants, and helped the seniors to rebuild social networks and gain more emotional support. Their circles of local friends needed to be rebuilt (Lin et al., 2020). These are beneficial to improve their physical and mental health and expand social network, and ultimately relieve anxiety. Second, we suggest that making elderly migrants promote resilience is an effective way to improve their life satisfaction. Third, proposing local registered residents treat elderly migrants more equally, and developing self-identity among elderly migrants. A better social, economic, and cultural environment can benefit internal migrants’ health status (Lin et al., 2016).

## Data availability statement

The raw data supporting the conclusions of this article will be made available by the authors, without undue reservation.

## Ethics statement

The studies involving human participants were reviewed and approved by the Institutional Review Board of Nanjing Medical University. The ethics committee waived the requirement of written informed consent for participation.

## Author contributions

YH: conceptualization, data curation, formal analysis, writing—original draft, and writing—review and editing. LZ, SY, and RD: investigation, formal analysis, and writing—review and editing. HW: formal analysis and writing—review and editing. WZ: conceptualization, investigation, and writing—review and editing. JY: conceptualization, supervision, writing—review and editing, and funding acquisition. All authors contributed to the article and approved the submitted version.
